# Developmental Hazard of Environmentally Persistent Free Radicals and Protective Effect of TEMPOL in Zebrafish Model

**DOI:** 10.3390/toxics9010012

**Published:** 2021-01-16

**Authors:** Xia Guan, Lisa Truong, Slawomir M. Lomnicki, Robyn L. Tanguay, Stephania A. Cormier

**Affiliations:** 1 Department of Environmental Sciences, Louisiana State University, Baton Rouge, LA 70803, USA; guanxiafrp@hotmail.com (X.G.); slomni1@lsu.edu (S.M.L.); 2 Sinnhuber Aquatic Research Laboratory, Department of Environmental & Molecular Toxicology, Oregon State University, Corvallis, OR 97333, USA; lisa.truong@oregonstate.edu (L.T.); robyn.tanguay@oregonstate.edu (R.L.T.); 3 Department of Biological Sciences, Louisiana State University, Baton Rouge, LA 70803, USA; 4 Pennington Biomedical Research Center, Baton Rouge, LA 70803, USA

**Keywords:** EPFRs, DCB-230, TEMPOL, zebrafish, antioxidant

## Abstract

Environmentally persistent free radicals (EPFRs) can be detected in ambient PM_2.5_, cigarette smoke, and soils and are formed through combustion and thermal processing of organic materials. The hazards of EPFRs are largely unknown. In this study, we assess the developmental toxicity of EPFRs and the ability of TEMPOL (4-Hydroxy-2,2,6,6-tetramethylpiperidine 1-oxyl) to protect against such hazards using zebrafish embryos. Particles containing EPFRs were acquired by dosing dichlorobenzene (DCB) vapor on the Cab-o-sil/5% CuO particles at 230 °C in vacuo (referred to as DCB-230). The particles were suspended in ultrapure water to make 1 mg/mL of stock solution from which series dilution was undertaken to obtain 10, 20, 30, 40, 50, 60, 80, and 100 µg/mL final test solutions, which were then placed in individual wells with a 4 h postfertilization (hpf) zebrafish embryo. Plates were run in duplicate to obtain a sample size of 24 animals per concentration; 12 embryos were exposed per concentration per plate. Statistical analysis of the morphology endpoints was performed. We investigated overt toxicity responses to DCB-230 in a 22-endpoint battery that included developing zebrafish from 24–120 hpf. Exposure to concentrations greater than 60 µg/mL of DCB-230 induced high mortality in the developmental zebrafish model. Exposure to EPFRs induced developmental hazards that were closely related to the concentrations of free radicals and EPFRs. The potential protective effects of TEMPOL against EPFRs’ toxicity in zebrafish were investigated. Exposure to EPFRs plus TEMPOL shifted the concentration to an induced 50% adverse effect (EC50), from 23.6 to 30.8 µg/mL, which verifies TEMPOL’s protective effect against EPFRs in the early phase of zebrafish development.

## 1. Introduction

Environmentally persistent free radicals (EPFRs) are primarily emitted from combustion and thermal processing of organic materials, in which the organic byproducts interact with transition metal-containing particles to form free radical particle pollutants in the cool and post-flame zones of combustion systems [1,2,3]. EPFRs can also be observed in ambient PM_2.5_ [4,5], cigarette smoke [6,7], and soils from superfund sites [8]. The particles containing EPFRs have been shown to cause adverse health effects [9] by triggering oxidative stress in biological systems [10,11,12,13,14,15,16]. Oxidative stress is a result of an imbalance of the concentrations of reactive oxygen species (ROS), such as superoxide anion radical (O_2_^•−^), hydrogen peroxide (H_2_O_2_), hydroxyl (^•^OH), alkoxyl (RO^•^), peroxyl (RO_2_^•^), and semiquinone (Q^•−^) radicals and antioxidants [17]. Excessive ROS oxidize DNA, proteins, and lipids and also damage cells by activating a redox signaling cascade that eventually leads to apoptotic cell death. Khachatryan, et al. [17] detected ROS generated by EPFRs of 2-monochlorophenol, associated with CuO/silica particles, and reported yields of ^•^OH, O_2_^•−^, and H_2_O_2_. Although several animal studies on the toxicity of EPFRs have shown that EPFRs may be a risk factor for cardiac toxicity in healthy individuals and individuals with ischemic heart disease [18], and that in-utero exposure to EPFRs decreases energy expenditure and damages skeletal muscle mitochondrial function in adult mice [15,16,19], we still understand little regarding how exposure during early life to EPFRs affects vertebrate development and how to attenuate EPFR toxicity.

Previous studies have demonstrated the pivotal role of free radical scavengers/antioxidants in inhibiting or reducing ROS, which causes inflammatory and neuropathic pain [20], especially TEMPOL (4-Hydroxy-2,2,6,6-tetramethylpiperidine 1-oxyl) and PBN (N-tert-butyl-α-phenylnitrone). TEMPOL is a cell-permeable free radical scavenger with unique antioxidant properties. It is a redox-cycling nitroxide that promotes the metabolism of many ROS and improves nitric oxide bioavailability [21]. TEMPOL degrades O_2_ via superoxide dismutase mimetic action, therefore inhibiting peroxynitrite’s formation. TEMPOL can suppress the formation of ^•^OH by preventing Fenton’s reaction catalyzed by transition metals. Multiple studies have shown that TEMPOL is effective in preventing the adverse consequences of oxidative stress and inflammation. For example, TEMPOL can ameliorate murine viral encephalomyelitis [22], protect against hypoxia-induced oxidative stress [23], alleviate intracerebral hemorrhage-induced brain injury in PC 12 cells [24], and ameliorate cardiac fibrosis in streptozotocin-induced diabetic rats [25]. The protective effect of the spin trap agent PBN on hyperoxia-induced oxidative stress has been studied [26]. Antioxidant treatment with PBN improves the cognitive performance and survival of aging rats [27]. PBN ameliorates the teratogenic effect of 5-azacytidine in rats [28]. PBN also shows neuroprotective effects [29]. We questioned whether TEMPOL or PBN would prevent EPFR-induced toxicity in a vertebrate biological system. The effects of TEMPOL and PBN against EPFRs’ developmental toxicity and the mechanism of protection remain to be clarified.

The zebrafish model has become popular in the field of toxicology and biomedical research due to its small size, high reproducibility, quick development, and the optically clarity of the embryo, which enables a quick assessment of developmental effects [30]. The cardiovascular, nervous, and digestive systems of zebrafish are similar to mammals. In addition, human and zebrafish genomes have a high level of resemblance, which makes it a feasible animal model for toxicological studies. Compared to the mouse model, zebrafish are cost- and space-effective and their developmental stages and life cycle are short. The zebrafish model has been successfully used to study the toxicity and bioactivity of nanoparticles, polycyclic aromatic hydrocarbons (PAHs) [31], 2,3,7,8-tetrachlorodibenzo-*p*-dioxin (TCDD) [32], pesticides [33], and more. To the best of our knowledge, there is no study assessing the toxicity of EPFRs and/or the effect of antioxidants using the zebrafish model.

The objectives of this work were (1) to establish a zebrafish model for studying whether exposure to EPFRs induces developmental toxicity and (2) to investigate if toxicity could be reduced by inhibiting the EPFR-mediated oxidative stress.

## 2. Materials and Methods

### 2.1. Chemicals

The chemicals 4-Hydroxy-2,2,6,6-tetramethylpiperidine 1-oxyl (TEMPOL, ≥97% nitrogen content, CAS 2226-96-2, Cat. No. 581500) and N-tert-butyl-α-phenylnitrone (PBN, ≥98%, CAS 3376-24-7) were purchased from Sigma Aldrich. Particles containing EPFRs were prepared as follows. The wetness impregnation method was applied to prepare Cab-o-sil/5% CuO particles [3]. EPFR-containing particles were acquired by dosing dichlorobenzene (DCB) vapor on the particles at 230 °C in vacuo. The samples were referred to as DCB-230.

Three controls were used in the experiments: Cab-o-sil particles dosed with DCB vapor at 50 °C in vacuo (Cab-o-sil-DCB); cab-o-sil/5% CuO particles; and water (vehicle control).

### 2.2. Zebrafish Embryo

Tropical 5D wild-type adult zebrafish were housed at an approximate density of 1000 per 100 gallons. Spawning funnels were placed into the tanks the night prior, and embryos were collected and staged as described in Kimmel et al. [34]. The studies were completed in accordance with protocols approved by the Oregon State University Institutional Animal Care and Use Committee (IACUC) (protocol approval number: 5113; approval date: 11 October 2018). To increase bioavailability, pronase (63.6 mg/mL, ≥3.5 U/mg) was used to enzymatically remove the chorion at 4 h postfertilization (hpf) using a custom automated dechorionator.

### 2.3. Zebrafish Embryo Assay

#### 2.3.1. Chemical Exposures

The concentration of DCB-230 was 4.83 × 10^16^ spins/g (the material contained 4.83 × 10^16^ radicals per gram). The samples were suspended in ultrapure water to make 1 mg/mL of stock solution from which series dilution was undertaken to obtain 10, 20, 30, 40, 50, 60, 80, and 100 µg/mL solutions for direct addition of 100 µL into wells of the 96-well test plate.

The eight-point dose concentration curve and the 96-well plate layout for the chemicals are shown in Table 1. Plates were run in duplicate to obtain a sample size of 24 animals per concentration; one embryo was exposed per well and 12 embryos were exposed per concentration per plate. All testing conditions were identical across replicate plates.

#### 2.3.2. Antioxidant Exposures

TEMPOL and PBN were used to test the effects of antioxidants to fight against free radicals. TEMPOL and PBN were each dissolved in 100% dimethyl sulfoxide (DMSO) to make respective 100 mM concentration stock solutions. Eight concentrations (0, 1, 2.54, 6.45, 16.4, 35, 74.8, and 100 µM) of TEMPOL or PBN in 0.1% DMSO were dispensed to the test plate by an HP D300 digital dispenser. Zebrafish developmental toxicity was evaluated under two exposure paradigms: static, nonrenewal exposure, and daily renewal exposure. For the daily renewal regimen, embryos with intact chorions were placed into individual wells of a 96-well plate and the exposure medium (with the same concentration of TEMPOL or PBN) was renewed daily until 120 hpf.

#### 2.3.3. EPFRs Plus Antioxidant Exposures

A static, nonrenewal exposure paradigm of antioxidants was applied. A total of 100 µM of TEMPOL in 0.1% of DMSO was dispensed to each of the EPFR solutions in the test plate by the HP D300, respectively. The concentration of freshly prepared DCB-230 was 1.53 × 10^17^ spins/g.

An internal QAQC (quality assurance and quality control) plate including 48 control animals and 48 animals exposed to 3,4-dichloroaniline (DCA) was run each day as an internal check for response consistency in animals from different hatches. For quality assurance, negative controls exhibited less than 20% cumulative mortality and morbidity and for the positive control, at least 80% of the DCA-exposed animals displayed adverse effects.

### 2.4. Embryo Photomotor Response Behavior

The embryos develop in the dark and when exposed to a strong flash of visible light at 28 hpf, the typical response is a burst of contralateral axial bending [35]. Photoreceptors in the hindbrain of zebrafish control this activity. Then, after the second flash of light, the response is recalcitrant. A 9 s interval was set between the two flashes of light. The assay consisted of 30 s of the dark background, then the first light pulse; after 9 s, the second pulse of light was applied, and finally 10 more seconds of dark. A white light emitting diode (LED) and infrared (IR) light were placed above the test plate; beneath the test plate mount, 850 frames of digital video were recorded at 17 frames s^−1^. Dead or malformed fish at the 24 hpf time point were excluded from the behavior data. The data were analyzed by statistical method [35].

### 2.5. Larval Photomotor Response Behavior

Zebrafish are free-swimming larvae at 120 hpf (five days post fertilization). Larval photomotor response tested total movement (swim distance) after exposing 120 hpf of zebrafish to multiple light -> dark transitions. In a 24-min analysis, we used Zebrabox (ViewPoint Life Sciences, Montreal, CA, USA) to track the total movement of larvae. High definition video was captured at 15 frames s^−1^. Using the manufacture’s software, data were analyzed in real-time. The 24-min analysis included four cycles of 3 min visible light and 3 min IR light (dark). The first light–dark cycle was for acclimation and the remaining three cycles were for statistical analysis. Dead or malformed fish at the 120 hpf time point were excluded from the behavior data. The area under the curve (AUC) was calculated for each concentration and a Kolmogorov–Smirnov test was used to determine if there was significance statistically. At *p* < 0.05 and a change in the AUC of >40%, significance was identified.

### 2.6. Statistical Analysis

*Morphology*. We used the R code to analyze the morphology endpoints data. The data used were binary incidences recorded for each endpoint from the Zebrafish Acquisition and Analysis Program (ZAAP). For each concentration and endpoint incidence, we plotted stacked observations to visualize the data. Compared to the control incidence rate, each chemical–endpoint pair reached a significant threshold. For each endpoint, binary data (0, 1) were recorded. Based upon the binomial test result, the significance threshold was estimated and visualized on the plots. Once the significance threshold was reached, we indicated the observation as red. No statistically significant effects were observed for the plate or well location among the controls (concentration = 0).

## 3. Results and Discussion

### 3.1. Mortality and Morphology Responses to EPFRs

**Figure 1:**
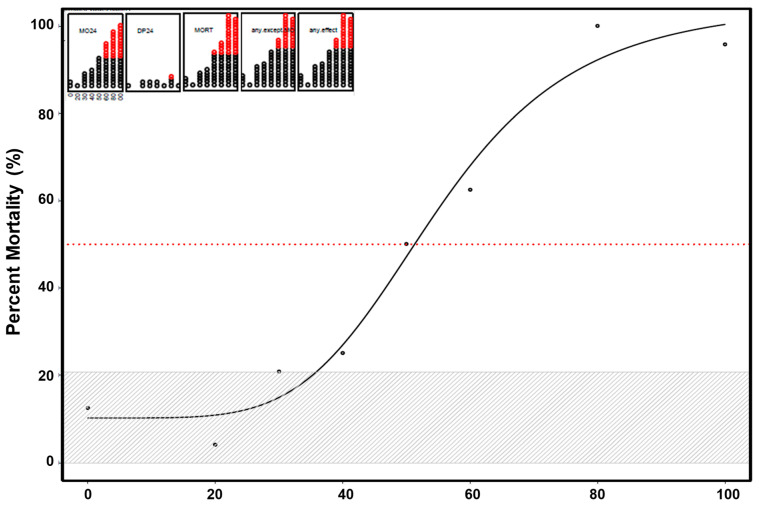
Incidence of overt toxicity responses to DCB-230 at 24 and 120 hpf. The top left inset is a depiction of 5 endpoints—two at 24 h (MO24 = mortality, DP24 = delayed development progression), and total mortality at five days (MORT). Two summary endpoints indicate individuals with any morbidity (any.except.MO), and any morbidity + mortality exhibited (any.effect). Each affected individual is illustrated as a circle and once a statistical threshold is passed, the individual is depicted as red. The logistic regression is shown over seven concentrations, with an EC50 of 54.8 µg/mL (red dotted line). The gray hash represents the two means’ standard deviations around the control. Statistical significance was determined using a Fishers Exact Test, *p* < 0.01.

Overt toxicity responses to DCB-230 in a 22-endpoint battery that included developing zebrafish from 24 hpf–120 hpf are summarized in Figure 1. Note that each observation of an endpoint corresponds to one data point on the endpoint graphs, and points in red indicate that the cumulative incidence of that endpoint, at a given concentration, reached statistical significance. Exposure to 60 µg/mL DCB-230 or above was associated with high mortality by 24 hpf. Figure 1 (inset) is offered as an “at-a-glance” synopsis of developmental toxicity across all samples, concentrations, and endpoints. Applying logistic regression to the data, an estimated concentration to cause a 50% effect (EC_50_) is 54.8 µg/mL with the concentration of EPFRs at 4.83 × 10^16^ spins/g.

### 3.2. Neurotoxicity of EPFRs

**Figure 2:**
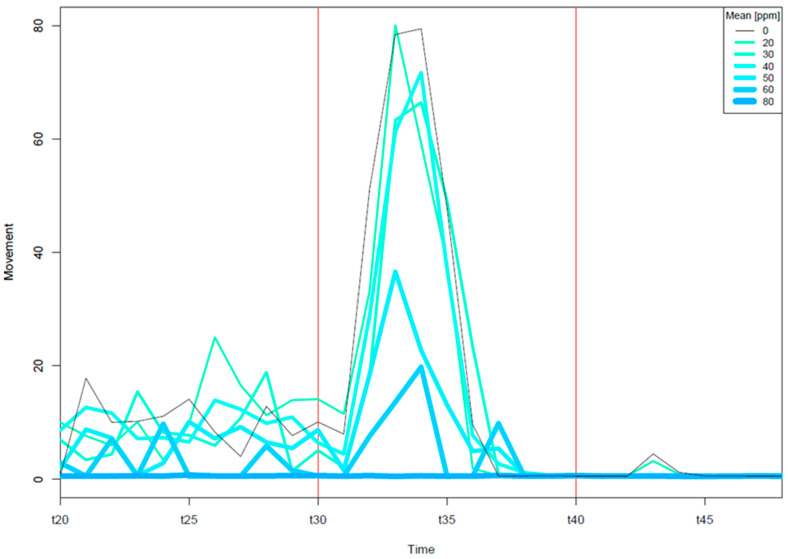
Embryonic photomotor response in embryos. Embryos were developmentally exposed and kept in the dark until 24 hpf. At 24 hpf, a typical photomotor response was exhibited (black line) with occasional movement in the dark and increases in excited movement after the pulse of light (red vertical line); there was little to no activity after the second pulse of light.

To test if EPFR exposures could affect early life stage behaviors, we performed two neurobehavioral assays. An embryo photomotor response (EPR) behavior assay was conducted at 24 hpf. Before the first flash of light, EPR typically exhibited limited basal activity; right after illumination, a burst of axis bending occurred. In response to the second light flash, there was little or no movement (Figure 2). As a response to the first light flash, with the DCB-230 solution at concentrations of 50 and 60 µg/mL, embryos were significantly hypoactive with fold changes ranging from 45 to 90% relative to the controls (K-S test, *p* < 0.05, delta > 40%). Considering the high incidences of mortality and morphology defects, the hypoactive EPR identified earlier in the same fish indicates that the EPR could predict future adverse outcomes. It does not directly suggest neurotoxicity, as many non-neuronal chemical effects could lead to hypoactivity. At 80 µg/mL, DCB-230 caused significant embryo mortality.

**Figure 3:**
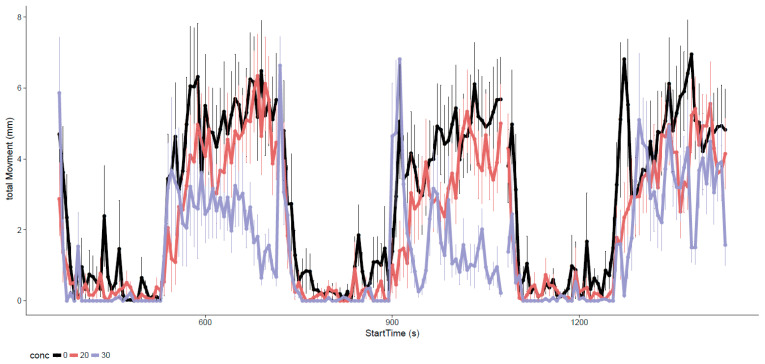
Larval photomotor response to alternating light and dark cycles after static exposure of DCB-230. Data are reported as mean ± standard deviation at each second time bin. Only concentrations that comprised 70% phenotypically normal animals are displayed.

At 120 hpf, we performed the second behavioral assay to investigate larval photomotor response (LPR) changes in swimming activity after a light–dark cycle. Excluding morphologically deformed animals, aberrant LPR in an otherwise normal animal was more likely to directly implicate neurotoxicity than the EPR. The typical LPR (Figure 3) showed modest movements in the light and obvious increases in motor activity in the dark. Compare to control fish, higher EPFR concentrations led to hypoactive swimming activity.

### 3.3. Mortality and Morphology Responses to Controls

**Figure 4:**
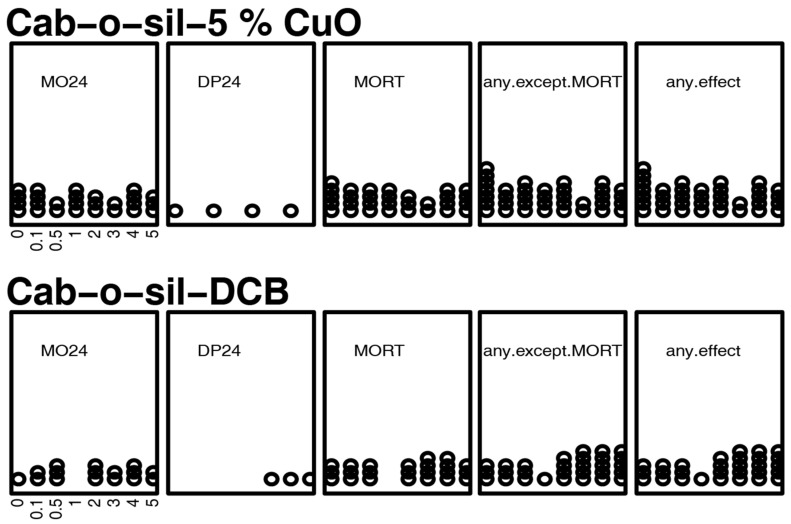
Incidence of overt toxicity responses to control DCB (at the concentrations of 0, 0.1, 0.5, 1, 2, 3, 4, and 5 µg/mL in ultrapure water) at 24 and 120 hpf. of the figure depicts five endpoints—two at 24 h (MO24 = Mortality, DP24 = delayed development progression), and total mortality at five days (MORT). Two summary endpoints indicate individuals with any morbidity (any.except.MO), and any morbidity + mortality exhibited (any.effect). Each affected individual is illustrated as a circle.

Overt toxicity responses to two controls (Cab-o-sil/5% CuO and Cab-o-sil-DCB) in a 22-endpoint battery that included developing zebrafish from 24 hpf–120 hpf are summarized in Figure 4. No significant results were observed. The third control was water (zebrafish embryo media).

### 3.4. Mortality and Morphology Responses to Antioxidants (TEMPOL or PBN)

If the toxicity of EPFR particles in zebrafish is caused by triggering oxidative stress in the biological systems, then it follows that during EPFR exposure, neutralizing EPFRs could reverse or significantly ameliorate the damage. We tested this hypothesis using the free radical scavenger PBN and TEMPOL because they have demonstrated protective effects in many rat models [36,37]. Before TEMPOL or PBN solutions were applied together with DCB-230, their individual effects on zebrafish embryos were tested. A total of eight concentrations were tested (0, 1, 2.54, 6.45, 16.4, 35, 74.8, and 100 µM) with 24 animals per concentration. Since antioxidants may lose effectiveness as time goes by, in addition to static exposure, we also studied daily renewal exposure by adding the same amount of antioxidants into each well from day 1 to day 4.

**Figure 5:**
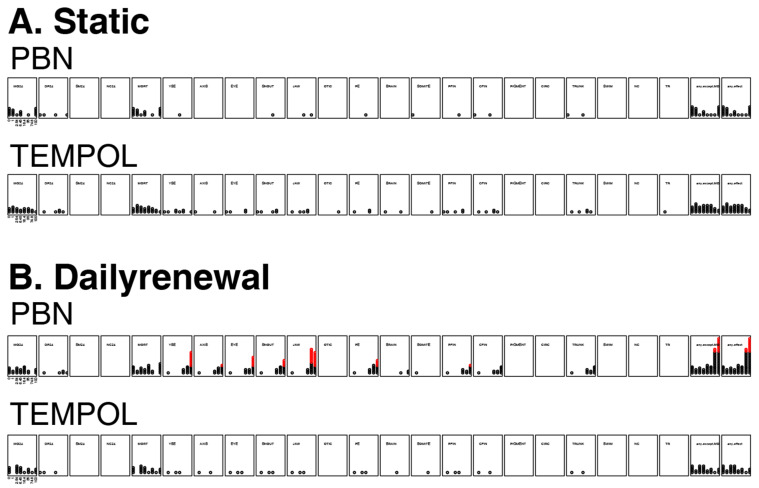
Incidence of overt toxicity responses to (**A**) static exposures and (**B**) daily renewal of PBN and TEMPOL at 24 and 120 hpf. MO24 = Mortality, DP24 = delayed development progression at 24 hpf, MORT = total mortality at five days. Two summary endpoints indicate individuals with any morbidity (any.except.MO), and any morbidity + mortality exhibited (any.effect). The other panels represent the morphological endpoints assessed. Each affected individual is illustrated as a circle and, once a statistical threshold is passed, the individual is depicted as red. YSE: yolk sac edema; AXIS: bent axis; EYE: abnormal eyes; SNOUT: abnormal snout; JAW: jaw; PE: pericardial edema and PFIN: pectoral fins.

Overt toxicity responses to PBN and TEMPOL in a 22-endpoint battery that included developing zebrafish from 24 hpf–120 hpf are summarized in Figure 5. Static, nonrenewal exposure of dechorionated embryonic zebrafish to both PBN and TEMPOL did not result in a statistically significant increase in mortality or any adverse outcome (Figure 5A). The daily renewal regime led to significant mortality and sublethal effects at 74.8 and 100 µM of PBN (Figure 5B). Static exposure of dechorionated embryonic zebrafish to both TEMPOL and PBN did not lead to a statistically significant increase in mortality or any adverse outcomes. Therefore, we chose static exposure of TEMPOL or PBN to study their potential protective effect against EPFRs.

### 3.5. Mortality and Morphology Responses to EPFRs Plus Antioxidants

**Figure 6:**
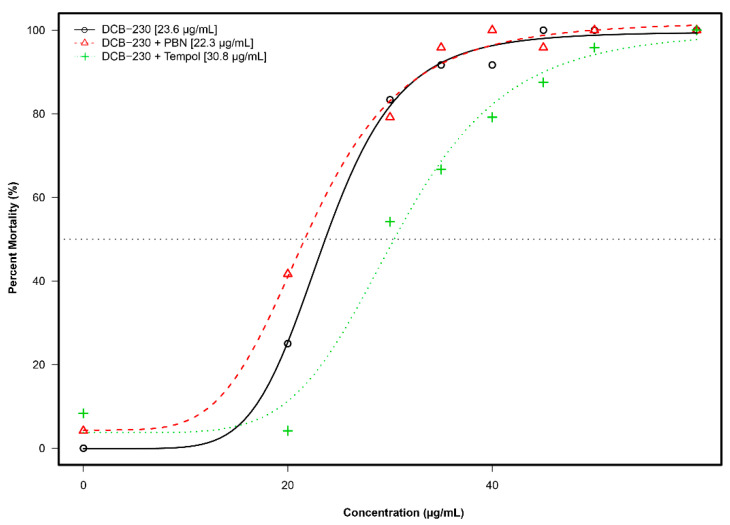
Incidence of overt toxicity responses to DCB-230 and antioxidants at 120 hpf. The modeling of the data illustrates that DCB-230 alone (black) and DCB-230 + PBN (red) had similar EC50s while DCB-230 + TEMPOL (green) was distinctly different.

Overt toxicity responses to EPFRs plus antioxidants in a 22-endpoint battery that included developing zebrafish from 24 hpf–120 hpf are summarized in Figure 6. TEMPOL shifted EC_50_ (µg/mL) of zebrafish from 23.6 (DCB-230) to 30.8 (DCB-230 plus TEMPOL) (Figure 6). PBN could not mitigate the toxicity of EPFRs; EC_50_ (µg/mL) slightly changed from 23.6 (DCB-230) to 22.3 (DCB-230 plus PBN).

Both TEMPOL and PBN are antioxidants. Tempol moderately extended survival in the zebrafish model presumably by inhibiting oxidative damage, while PBN did not. TEMPOL is a cyclic nitroxide with multiple functions that displays low toxicity in vivo [38]. This small-molecule cell-permeable superoxide dismutase (SOD) mimetic is able to act as SOD1, SOD2, SOD3, and catalase in vivo. DCB-230 was generated from free radical precursor DCB and also induced a mixture of free radicals, including ^•^OH, O_2_^•−^, and H_2_O_2_. TEMPOL’s significant ability to inhibit or eliminate oxidative stress induced by superoxide free radicals ameliorated toxicity of EPFRs in zebrafish development. Compared to TEMPOL, PBN was not cell permeable, which might have mitigated its effects against oxidative stress induced by EPFRs. The evidence for PBN’s long-term mixed effects is supported by Kubová’ s work in which they concluded that PBN’s effects were both beneficial and detrimental at the time of status epilepticus in juvenile rats [39].

## 4. Conclusions

Exposure to DCB-230 was developmentally hazardous in the zebrafish model, which elicited a series of developmental abnormalities included yolk sac edema and abnormalities of the axis, eye, snout, jaw, trunk, swimming bladder, etc. Adverse behavioral effects were also observed. The toxicity of EPFRs is closely related to the concentration of free radicals and EPFR suspension. TEMPOL ameliorates the toxicity of DCB-230. The EC50 of zebrafish mortality after exposures to EPFRs and EPFRs plus TEMPOL changed from 23.6 to 30.8 µg/mL. PBN could not alleviate the toxicity of EPFRs in the zebrafish model. TEMPOL is considered as a membrane-permeable agent and it could directly react with free radicals generated in the system. We believe that TEMPOL suppresses the toxicity of EPFRs by preventing oxidative stress and inflammation in intact zebrafish embryos. Further experiments should be conducted to verify that TEMPOL has better permeability than PBN in zebrafish. Although the detailed mechanism of TEMPOL’s fight against EPFRs needs further study, our current findings pave the way for further studies of the protective effects of cyclic nitroxides in developmental disease animal models.

## Figures and Tables

**Table 1:** DCB-230 concentrations (µg/mL) and the plate layout in 96 wells.

	1	2	3	4	5	6	7	8	9	10	11	12
A	100	100	100	100	100	100	100	100	100	100	100	100
B	80	80	80	80	80	80	80	80	80	80	80	80
C	60	60	60	60	60	60	60	60	60	60	60	60
D	50	50	50	50	50	50	50	50	50	50	50	50
E	40	40	40	40	40	40	40	40	40	40	40	40
F	30	30	30	30	30	30	30	30	30	30	30	30
G	20	20	20	20	20	20	20	20	20	20	20	20
H	0	0	0	0	0	0	0	0	0	0	0	0

## Data Availability

Data is contained within the article.
